# Dynamic ultraviolet harmonic beam pattern control by programmable spatial wavefront modulation of near-infrared fundamental beam

**DOI:** 10.1515/nanoph-2023-0300

**Published:** 2023-06-23

**Authors:** Seungjai Won, Seungman Choi, Taewon Kim, Byunggi Kim, Seung-Woo Kim, Young-Jin Kim

**Affiliations:** Department of Mechanical Engineering, Korea Advanced Institute of Science and Technology, 291 Daehak-ro, Daejeon, Republic of Korea

**Keywords:** coherent frequency upconversion, dynamic beam shaping, harmonic generation, near-infrared, spatial wavefront modulation, ultraviolet

## Abstract

The ultraviolet (UV) wavelength regime is attracting increasing attention because of its growing demand in semiconductor lithography, spectroscopy, and imaging applications owing to its high spatial resolution and high photon energy. However, beam shape control, beam delivery, and wavefront manipulation of UV laser beams usually require highly dedicated optics because of the strong UV absorption of most optical materials and the high surface precision required for tailoring short wavelengths, thus limiting a broader application of UV wavelengths. Here, we demonstrate a novel dynamic UV harmonic beam pattern control by manipulating the near-infrared (NIR) wavefront of the fundamental wavelength of a femtosecond pulse laser. The temporal and spatial coherences in an optical harmonic generation are known to be well preserved. Therefore, the spatial beam distribution of UV harmonic beams (*λ* = 400 and 266 nm for second and third harmonics, respectively) could be readily controlled by tailoring the wavefront of the driving infrared (IR) beam, and this approach can be expanded to higher-order harmonics in the vacuum ultraviolet (VUV) or extreme ultraviolet (EUV) regimes. Moreover, this enables fast polarization-sensitive UV beam switching at a speed of 6.7 frames/s in a depth-resolving manner. To efficiently separate the UV beam from the strong fundamental IR background beam, a non-collinear harmonic generation configuration is introduced. This facile dynamic UV beam control technique enables arbitrary wavefront control of UV laser beams for high-precision laser patterning, polarization-sensitive encryption, and 3D holograms.

## 1 Introduction

As ultraviolet (UV) light sources emit high photon energies (3.1–6.2 eV) and have high spatial resolutions, they are in high demand in semiconductor photolithography [1], atomic spectroscopy [2], high-resolution imaging [3], and high-precision metrology [4]. The introduction of UV and extreme ultraviolet (EUV) laser beams enabled the higher-density integration of microelectronics [5] and the measurement of nanoscale defects [6] over the past few decades. Moreover, UV-based optical data storage or encryption [7, 8] has attracted considerable attention owing to its high spatial frequency and security. Meanwhile, UV light is easily absorbed by most materials because of its high photon energy [9]. These properties lead to a lack of a solid-state gain medium for UV laser oscillation [10] and control optics in the UV wavelength range [11]. Especially, for beam delivery and spatial adjustment of the wavefront or amplitude of the UV wavelength, continuous efforts have been taken to develop metal-coated reflecting optics [12] and specifically designed diffractive elements [13]. Recently, several schemes have been proposed based on nonlinear harmonic generation to achieve a handy UV beam manipulation, and these schemes led to coherent light emission at integer-multiple optical frequencies from those of the input driving beam [14]. For example, introducing a diffractively structured nonlinear optical material, such as a metasurface, can enable the manipulation of the spatial properties of UV harmonics from an infrared (IR) laser beam in the harmonic generation step without UV control optics [15].

Nonlinear harmonic generation enables the transfer of spatiotemporal information of the fundamental beam to the harmonic waves at shorter wavelengths. In other words, the properties of the generated short-wavelength light such as complex amplitude [16] and polarization [17] can be modulated by tailoring the wavefront of the fundamental input beam. This is possible because the spatial and temporal phase coherences of the input laser beam are mostly preserved during the coherent frequency upconversion process under atomic scale light–matter interactions [14, 18, 19]. In particular, this coherent process has more advantages with a high-coherence pulse laser than with a broadband lamp, as it provides a well-controllable high-order harmonic ultrafast EUV pulse that can be used for ultrafast optical applications with attosecond-scale timing resolutions [20]. Additionally, in this harmonic generation, common adaptive optics in the IR regime with a spatial light modulator (SLM) can dynamically deliver optical properties to shorter wavelengths [21], which cannot be achieved with a pre-patterned diffractive nonlinear medium or metasurfaces. Using these active devices can also facilitate the polarization-dependent modulation of UV beams, as frequently reported in the case of the visible (VIS) – IR regime [22]. Although this fundamental beam modulation-based harmonic generation can dynamically manipulate the beam shape of the UV light and pulses with a lower wavelength, it has not been demonstrated yet.

In this work, we demonstrate an SLM-based dynamic beam shaping of UV harmonics through programmable spatial phase modulation of the driving beam. The spatial beam distribution of the generated UV harmonics was manipulated by tailoring the wavefront of the driving infrared (IR) beam and focusing it on a nonlinear crystal. Two sets of driving IR beams were non-collinearly overlapped on a quartz crystal with an appropriate crossing angle, allowing the generated UV harmonic to have a different propagation direction from that of the driving IR beam. Therefore, a spectral filter was not required for IR blocking, which could cause a significant decrease in the intensity of the generated UV harmonics. In addition, this could prevent unwanted sample or filter damage issues because of the strong IR driving beam. The propagation direction of the generated harmonic waves is simply determined by the momentum-conservation condition of the driving beam. Based on this configuration, fast UV pattern switching using the polarization-sensitive diffraction behavior of the SLM was demonstrated. By appropriately adjusting the polarization state of the driving beam and selectively modulating the two beams at the SLM, the generated UV harmonic wave was changed into two different patterns. Each polarization-sensitive UV harmonic pattern can be generated at two different focal points or dynamically switched at a speed of 6.7 frames/s. Finally, the spatial modulation of deep-UV third harmonics (*λ* = 266 nm) with a MgO crystal was demonstrated, suggesting an efficient method for dynamically controlling the deep ultraviolet (DUV) or a shorter wavelength regime. This dynamic control of harmonic waves enables the facile wavefront manipulation of a UV laser beam for high-precision laser patterning, polarization-sensitive optical encryption, and 3D holograms.

## 2 Dynamic UV harmonic beam pattern control

### 2.1 Experimental configuration

**Figure 1:**
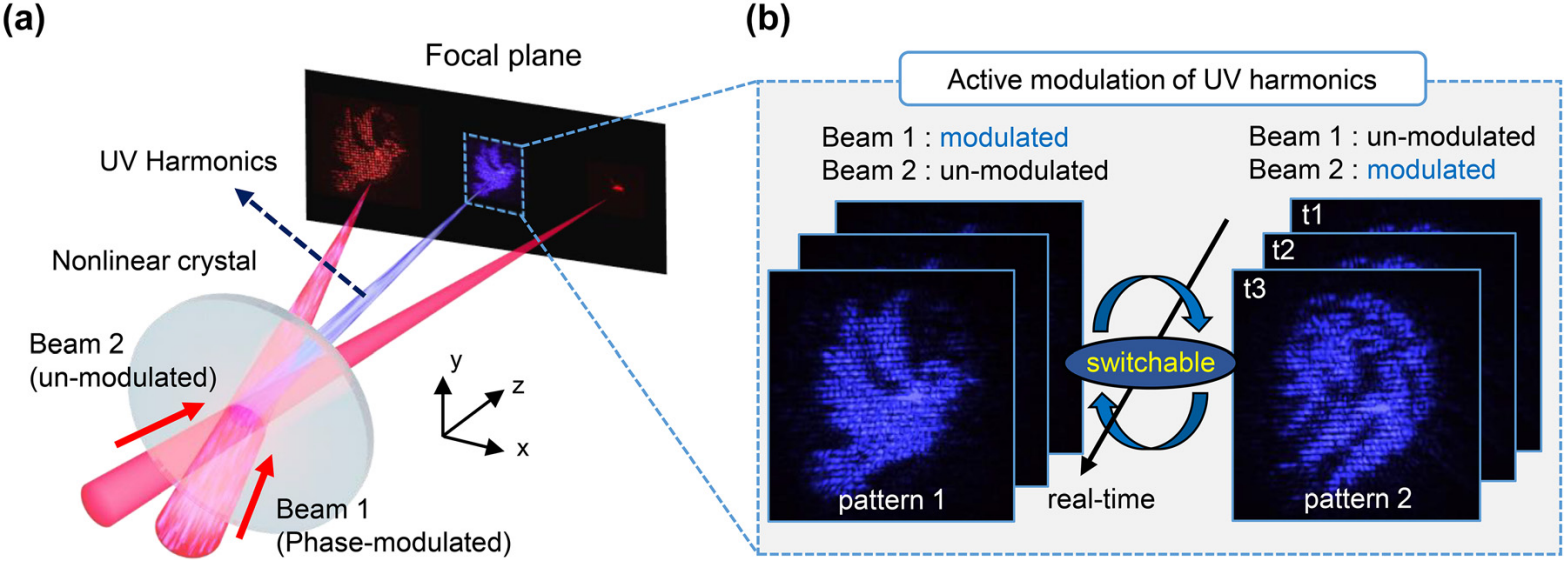
Conceptual illustration of dynamic harmonic UV beam control. (a) Non-collinear harmonic generation scheme and generated harmonic beam pattern at the focal plane. The phase-modulated and un-modulated driving IR pulses are non-collinearly irradiated into the nonlinear medium, generating harmonic UV pulses that are spatially shaped at the focal plane. (b) Dynamically switchable UV harmonic beam pattern by selectively modulating one of the two driving beams, or through real-time driving beam modulation.

Figure 1 illustrates the active UV modulation principle based on harmonic generation. Two driving IR femtosecond laser beams with different propagation directions are non-collinearly overlapped at the nonlinear optical quartz sample, thereby generating second-harmonic waves (Figure 1(a)). As the nonlinear optical harmonic generation is based on the coherent frequency upconversion process, the spatiotemporal information of the fundamental wavelength is transferred to the second-harmonic wave. When the overlapping position of the two beams is set before the focal point, the generated harmonics can be focused directly on the converging wavefront of the driving beam. The propagation direction of the harmonic waves depends on the momentum-conservation theorem. By simply modulating the spatial phase of the driving beams using an SLM, the harmonic waves at the focal plane can be controlled without any UV optical components. The pattern of harmonics can be further switched by selectively modulating one of the two driving beams (Beams 1 and 2 in Figure 1(a)), and each design can be controlled in real-time (Figure 1(b)).

**Figure 2:**
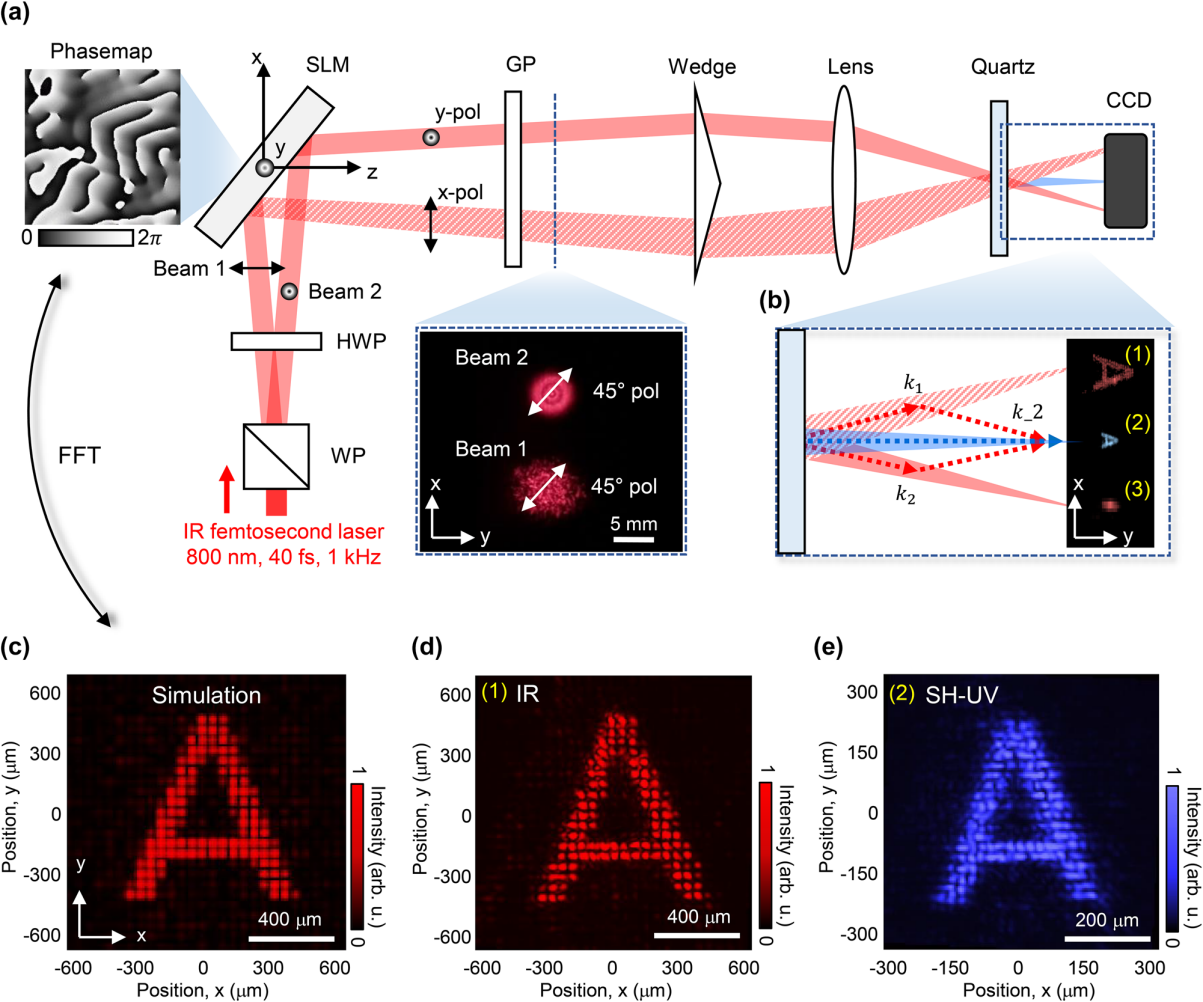
Configuration of the second harmonic beam modulation. (a) Experimental setup for non-collinear harmonic generation and control. (b) Wave vector of the second harmonic beam and captured image at the focal plane. (c) Simulation results for pattern ‘A’ based on Fresnel propagation. (d) Acquired image of driving beam pattern ‘A’ at the focal plane. (e) Acquired image of second harmonic UV pattern ‘A’ at the focal plane.

The detailed configuration based on this concept is shown in Figure 2. The entire system is composed of quasi-common path non-collinear harmonic generation [16], thereby becoming resistant to phase-shift caused by disturbance or path differences between two driving beams. The driving beam (having an 800 nm center wavelength, 40 fs pulse duration, 1 kHz repetition rate, 400 mW average power, and a Gaussian beam profile) was split into two beams of mutually orthogonal polarization by a Wollaston prism (WP). The polarization of the two beams was then adjusted horizontally (*x*-pol in Figure 2(a)) and vertically (*y*-pol in Figure 2(a)) using a half-wave plate (HWP). When these two beams were incident on different areas of the SLM (X13138, 1272 × 1024 pixels with 12.5 μm pixel pitch, Hamamatsu, Japan) with two different phase maps, only one beam (beam 1 in Figure 2(a)) with the horizontal polarization (*x*-polarization: the working polarization of the SLM) was phase-modulated, whereas the other beam with vertical polarization (beam 2 in Figure 2(a)) remained un-modulated. The phase-modulated beam produced the desired amplitude distribution at the focal plane. The two driving beams reflected from the SLM were set to the same linear polarization (45°) state using a Glan laser polarizer (GP), and the time delay between the two beams was matched using a wedge plate. These two beams were focused using a plano-convex lens (*f* = 200 mm, LA1253-B, Thorlabs, USA) and spatiotemporally overlapped at 50 mm before the focal point with a crossing angle of 5°. The nonlinear quartz crystal sample (200 μm thick, Biotain Crystal Co., Ltd., China) was placed at this overlapping position, thereby producing an interference pattern on the surface, as shown in Eq. S(1) in Section 1 of the Supplementary material. Generally, the harmonics emitted from non-collinear configurations can be described by phase matching conditions [23]. Compared to the gas medium, the phase mismatching due to the atomic dipole phase can be significantly decreased in the solid medium since the interaction layer is relatively short due to strong reabsorption [16]. Therefore, the wavefront of the driving beam is well preserved at the nonlinear surface, allowing the interference pattern to be well created at the surface. A detailed explanation is described in Section 2 of the Supplementary material. Based on the interference pattern on the nonlinear surface, a spatially modulated second harmonic UV (SH-UV) wave (*λ* = 400 nm) can be generated and diffracted from the wave model of non-collinear harmonic generation [24]. This determines the propagation direction and shape of harmonic waves at the focal plane. In this case, the un-modulated driving beam (beam 2 as seen in Figure 2(a)) operates as a zero-order beam; therefore, only the modulated driving beam (beam 1 in Figure 2(a)) affects the complex amplitude of the second harmonic. It should be noted that the propagation characteristics of the harmonic waves in a non-collinear irradiation can also be defined by the momentum conservation of two driving beams based on the photon model [24] (refer to the end of this part and Section 3 of the Supplementary material). The output power and nonlinear conversion efficiency of SH-UV pattern was 1.6 μW and 1.0 × 10^−3^ %. The details of output power and conversion efficiency are explained in Section 4 of the Supplementary material.

A UV-enhanced charge-coupled device (CCD) camera (CM-140MCL-UV, 1392 × 1040 pixels, 4.65 μm pixel pitch, JAI Co., Ltd., Denmark) was used to image the beam pattern of the driving beams and harmonics at the focal plane. To acquire the second harmonic signal exclusively without the fundamental beam, a stainless-steel IR block and bandpass filter (FB400-10, Thorlabs, USA) were installed in front of the CCD. Figure 2(b) shows the wave vector of the second harmonic beam and the captured image at the focal plane. In the image, (1) ‘A’ pattern of driving IR, (2) SH-UV, and (3) un-modulated driving IR are shown. Figure 2(d) and (e) show enlarged images of (1) and (2), respectively. In the case of the driving beam, the shape and size of pattern ‘A’ are consistent with the simulation result based on Fresnel propagation, as shown in Figure 2(c). The discrete dot pattern, which can be considered as a spatial frequency component owing to the pitch of the unit phase map array at the SLM, was generated both in the simulation and experiment. More details are given in Section 2.2. The calculated and measured intervals between the dot patterns were 50.0 and 48.7 μm, respectively. It should be noted that the dot interval can vary according to the focal length of the lens or the size of the unit phase map. Regarding the second harmonic shown in Figure 2(e), the generated pattern takes over the driving beam pattern, which is two times smaller than that of the driving beam. The measured dot interval of the SH-UV pattern was 24.2 μm, which is half of the driving beam pattern. This can be explained well by both the wave and photon models in the context of non-collinear harmonic generation. First, in the wave model, the interference pattern generated on the sample surface consists of a scale corresponding to the wavelength of the IR beam. The generated SH-UV is diffracted from this interference pattern of the IR driving beams. Since SH-UV has a two-times shorter wavelength than that of the IR driving beam, its propagation angle is defined as twice smaller than that of the driving beam according to the grating equation sin *θ* = *mλ*/*d*, where *θ* is diffraction angle, *m* is the diffraction order, and *d* is period of driving beam interference pattern. Therefore, the second harmonic wave at the focal plane is formed in a two-fold smaller pattern. Second, according to the photon model (Eq. S(3)), the propagation vector of the second harmonic photon generated by the two IR photons is at half the angle between the two driving beams which satisfies momentum conservation. This relationship works over the entire cross-sectional area of the second harmonic photon generation. As a result, the pattern size becomes half the original size at the focal point. Note that many experimental control factors can be considered in the formation of the UV harmonic pattern. For example, the power and pattern quality of the UV harmonics vary according to the power ratio of the two driving beams or the tilt and overlapping position of the nonlinear sample. The details are described in Sections 5 and 6 of the Supplementary material.

### 2.2 Phase-map generation for SLM via Fraunhofer diffraction theory

The relationship of the complex amplitude between the far field (at SLM) and the focal plane (at CCD) can be represented using the Fraunhofer diffraction theory, as shown in Eq. (1).

(1) $$\begin{array}{ll} g\left(x,y\right) & =h_{0}\exp\left(-j\pi \frac{x^{2}+y^{2}}{\lambda d}\right)\int \overset{\infty }{\underset{-\infty }{\int }}f\left(x^{\prime },y^{\prime }\right)\exp\\ & \;\times \left(j2\pi \frac{xx^{\prime }+yy^{\prime }}{\lambda d}\right)dx^{\prime }dy^{\prime } \end{array}$$

where *f*(*x*′, *y*′) and $g\left(x,y\right)$ represent the electric fields at the far field and focal plane, respectively. The integral part is the Fourier transform of *f*(*x*′, *y*′), represented as *F*(*v*_*x*_, *v*_*y*_), where *v*_*x*_ and *v*_*y*_ denote the spatial frequency at the focal plane ( $v_{x}=\frac{x}{\lambda d}$ and $v_{y}=\frac{y}{\lambda d}$ ). *λ* and *d* represent the wavelength of light and distance from the far field to the focal plane, respectively. Based on this relation, we designed a phase map (composed of 0–2π phase distribution) in the SLM plane using the Gerchberg–Saxton (GS) algorithm based on iterative Fourier transform [25], assuming that the input beam had Gaussian-shaped amplitude. To improve the diffraction efficiency for a given beam diameter (5 mm), an appropriately sized unit phase map (256 × 256 pixels) was input as an array with a 5 × 4 replica. It should be noted that this type of phase-map array can generate discrete dot patterns at the focal plane, thereby allowing the analysis of the spatial frequency depending on the wavelength. The size reduction of the second harmonic pattern in Figure 2 can also be explained by the Fraunhofer diffraction theory [26]. The distance between the harmonic emission point and the focal plane (50 mm) is sufficiently large when compared to the calculated Rayleigh length (3.14 mm) and the Fraunhofer distance (2 mm) of the second harmonic wave near the focal plane. Therefore, the harmonic generation plane can be regarded as a far field, and its propagation can be explained by the Fraunhofer theory. When the wavelength was reduced from 800 to 400 nm, the spatial frequencies (*v*_*x*_ and *v*_*y*_) at the focal plane were doubled; thus, a two-fold smaller pattern was generated.

### 2.3 Polarization-sensitive fast UV beam modulation

**Figure 3:**
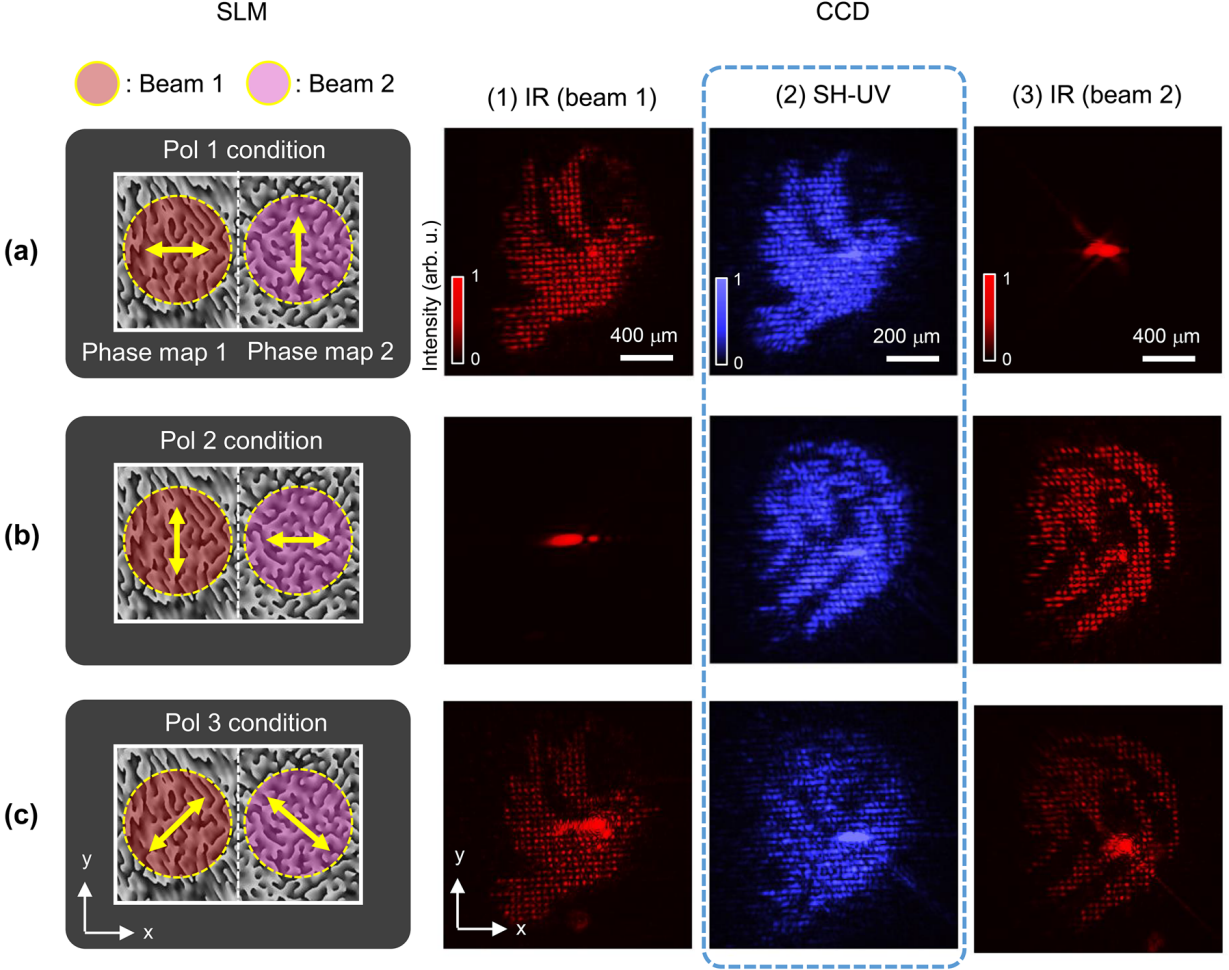
Polarization-dependent pattern switching of SH-UV. The left column shows the input phase map and polarization state of the two incident driving IRs on the SLM (plane 1). The figures on the right represent the acquired beam pattern at positions (1), (2), and (3) of the CCD (plane 2) shown in Figure 2. (a) Pol 1 condition and the generated IR/second harmonic UV ‘Pattern 1’. (b) Pol 2 condition and the generated IR/SH-UV ‘Pattern 2’. (c) Pol 3 condition and the generated SH-UV ‘pattern 3’, which is the superposition of patterns 1 and 2.

Figure 3 shows the modulation of SH-UV according to the polarization state of the two driving beams. The experimental configuration was the same as that shown in Figure 2, except for the phase map and polarization states of the driving beam. On the left and right sides of the SLM plane, phase maps 1 and 2 were input to generate the ‘bird’ and ‘tiger’ patterns, respectively. For the two driving beams incident on the SLM, we set three polarization conditions, denoted as ‘pol 1’, ‘pol 2’, and ‘pol 3’. Pol 1, 2, and 3 indicate that the polarization states of the driving beams 1 and 2 incident on the SLM are (0°, 90°), (90°, 0°), and (45°, 135°), respectively, as shown in Figure 3(a)–(c). In Figure 2(a), 0° is the *x*-polarization state, which is the working direction of the SLM. Based on these three polarization states, the beam shape of each driving beam and/or SH-UV was acquired at the focal plane, as shown on the right side of Figure 3. The three columns of the images are (1) IR driving beam 1, (2) SH-UV, and (3) IR driving beam 2 from the left. In the pol 1 condition, as shown in Figure 3(a), beam 1 was modulated in a ‘bird’ pattern, whereas beam 2 was un-modulated and reflected in the 0th-order. Next, in the pol 2 condition shown in Figure 3(b), beam 2 was modulated in a ‘tiger’ pattern, and beam 1 was un-modulated. This result is attributed to the polarization dependence of the SLM, as mentioned earlier. Only a wave with the same polarization as the working direction has a high diffraction efficiency, producing the desired beam pattern. The beam that has a polarization perpendicular to the working direction of the SLM is almost reflected as a 0th-order diffraction component. Under the pol 3 condition, as shown in Figure 3(c), both the driving beams are modulated because both beams have a polarization component in the working direction of the SLM. The pattern shape of the SH-UV was determined based on the modulated driving beam. In the pol 1 condition, SH-UV followed the shapes of beam 1 and was generated in the form of a ‘bird’ shape. On the other hand, in the pol 2 case, SH-UV was generated in the ‘tiger’ shape, following beam 2. Note that in the pol 3 case, the SH-UV pattern was generated with overlapping of ‘bird’ and ‘tiger’ shapes, because both modulated beams 1 and 2 were involved in determining the shape of harmonics. The reduced pattern size and increased spatial frequency of SH-UV can be sufficiently explained by the aforementioned momentum conservation or interference pattern at the surface of the crystal, as shown in Figure 2. The SH-UV pattern was formed within a size of approximately 625 × 765 μm. Considering SH-UV’s dot interval of 24.2 μm, an image is composed of approximately 26 × 31 dot arrays. The significance of this result is that the spatial shape of the SH-UV can be modulated simply by rotating the polarization of the two IR beams. Based on this configuration, we conducted dynamic harmonic beam modulation (Supplementary videos 1–3) using the real-time phase-map controllability of SLM. The video clips were obtained under the pol 1, 2, and 3 conditions, respectively. Under the pol 1 and 2 conditions, SH-UV with different shapes corresponding to beams 1 and 2, respectively, were modulated in real-time. Under the pol 3 condition, the superimposed SH-UV pattern, which had two shapes generated from beams 1 and 2, was controlled dynamically. These videos show that real-time SH-UV beam modulation can be further controlled depending on the polarization states of the driving beam. The frame rate of the video was set to 6.7 Hz considering the rising time (30 ms) and falling time (80 ms) of the liquid crystal inside the SLM in our current research. A higher switching speed of a few kHz or even more can be achieved by using a MEMS-based light modulator [27].

**Supplementary video 1:** 

**Supplementary video 2:** 

**Supplementary video 3:** 

**Figure 4:**
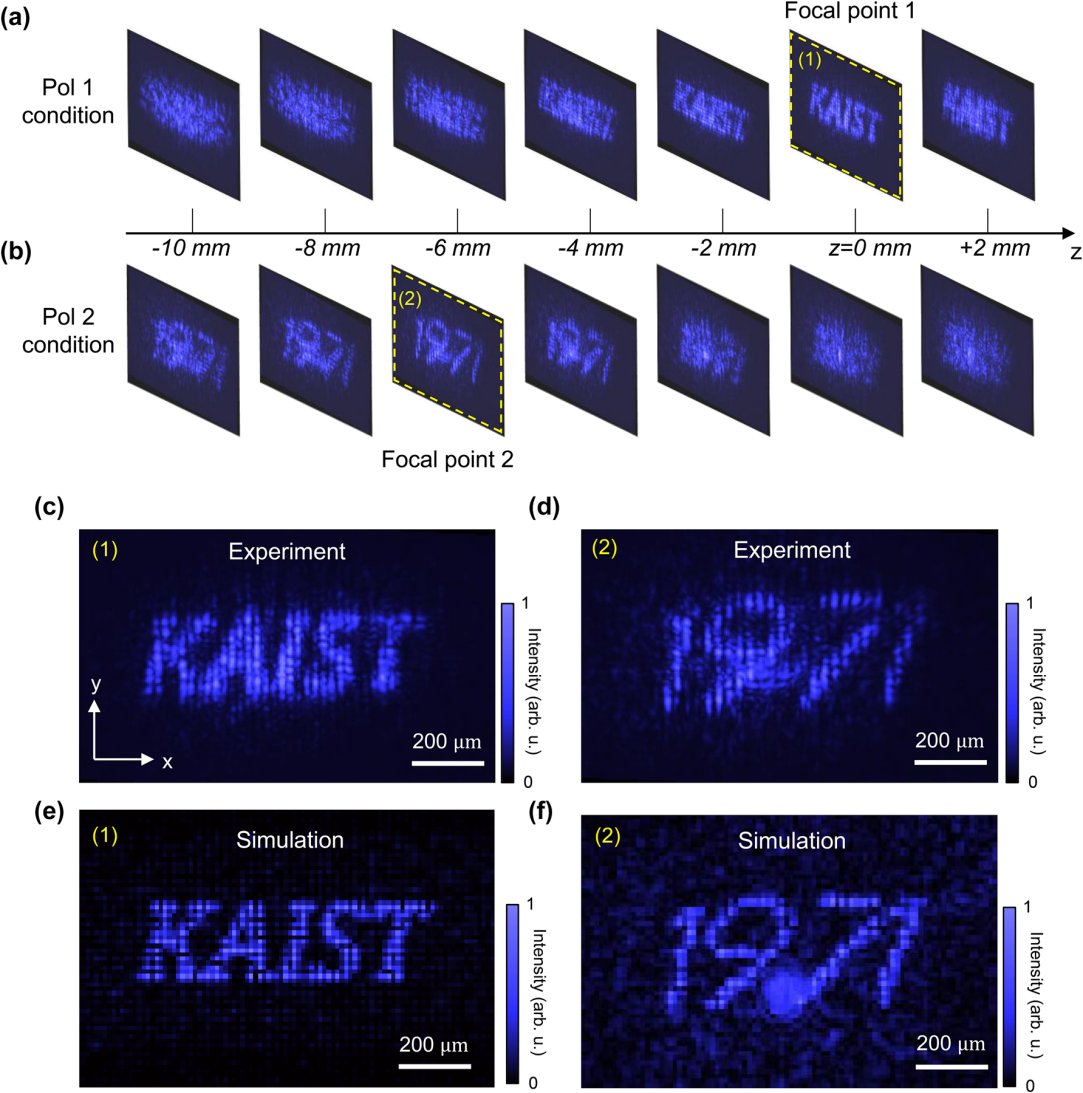
Generation of SH-UV pattern with the different focal points. (a) Propagation of the ‘KAIST’ pattern through optical axis under pol 1 condition, shown in Figure 3(a). (b) Propagation of 6-mm-defocused ‘1971’ pattern through optical axis under pol 2 condition, shown in Figure 3(b). (c–d) Detailed images at each focal point. (e–f) Simulation results of harmonic beam pattern at each focal point.

Based on the polarization-sensitive beam modulation of harmonic UVs, we demonstrate harmonic beam shaping with dual foci with respect to the polarization states of the driving beam, as shown in Figure 4. Phase maps 1 and 2 were input to the left and right sides of the SLM, respectively, to generate the letters ‘KAIST’ and ‘1971’. In phase map 2, a Fresnel lens pattern with a focal length of 4000 mm was additionally applied to induce defocusing. To image the SH-UV in a different plane, the CCD was mounted on a linear stage (DDS100/M, Thorlabs, USA) and scanned along the optical axis with intervals of 1 mm. Figure 4(a) shows an acquired SH-UV image in each plane under the pol 1 condition. The phase-modulated SH-UV wave was formed in a ‘KAIST’ pattern at focal point 1 (*z* = 0). On the other hand, under the pol 2 condition, the ‘1971’ SH-UV pattern was generated at different focal points at *z* = −6 mm, owing to the applied Fresnel lens function in phase map 2. Therefore, different UV patterns could be generated at different focal points along the optical axis. Figure 4(c) and (d) show an enlarged image at the focal point according to each polarization condition. In the ‘1971’ pattern, the interval of the dot (29.1 μm) is slightly larger than that of the ‘KAIST’ pattern (24.2 μm) at the focal plane. This is attributed to Fresnel propagation. To analyze the defocusing characteristics, we calculated the beam pattern at each focal point based on Fresnel propagation, with the assumption of the single-beam irradiation along the optical axis. The simulation results shown in Figure 4(e) and (f) are consistent with the experimental results, except for a slight pattern degradation that occurred because of non-collinear irradiation.

## 3 Third harmonic DUV beam shaping

**Figure 5:**
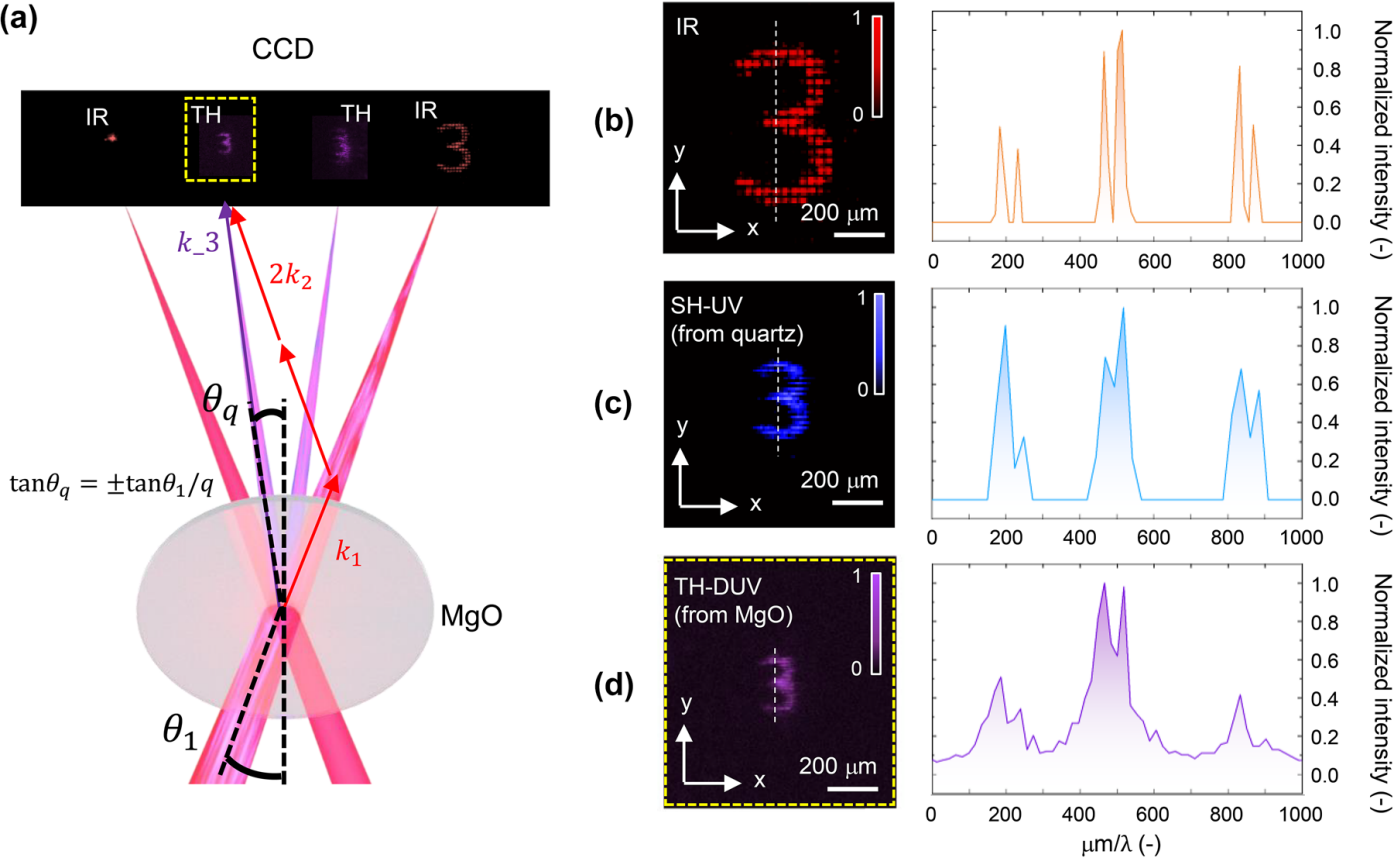
Third harmonic (*λ* = 266 nm, TH-DUV) beam modulation using MgO crystal. (a) Configuration of momentum conservation and divergence angle for third harmonic generation and acquired CCD images. (b–d) Generated pattern ‘3’ in (b) IR, (c) SH-UV, and (d) TH-DUV, respectively. The graphs on the right show the cross-sectional intensity distribution of the white dotted lines indicated in the images, divided by the wavelength.

Figure 5 shows the beam control of the third harmonic DUV (TH-DUV) with a MgO crystal (150 μm thick, <100>, Biotain Crystal Co., Ltd., China). To generate the third harmonic wave, three photons of the driving beam are required. According to the momentum conservation described in Section 3 of the Supporting Information, there are four possible cases for (*q*_1__,_ *q*_2_) = {(0,3),(3,0),(1,2),(2,1)} in the third harmonic generation. Among them, we considered only two cases: (1,2) and (2,1); these harmonics propagate in a direction different from that of the driving beam. To efficiently generate higher harmonic orders, a higher driving beam intensity is required. Therefore, the power of the driving beam was set to 1200 mW, which was three times higher than that of the second harmonic generation. A phase map was input into the SLM to generate the number ‘3’. The Γ–*K* direction of the MgO crystal was set to 0° relative to the *x*-coordinate. The polarization of the two driving beams was set to that of the pol 1 or pol 2 conditions. A 266-nm bandpass filter (10LF10-266, Newport, USA) was installed in front of CCD to acquire only the third harmonic signal. Figure 5(a) shows the propagating directions of generated third harmonics and captured images of third harmonic patterns. In the wave and photon model [24], the smallest divergence angle of the generated harmonic wave with a linear polarized driving beam *θ*_*q*_ follows the trend tan *θ*_*q*_ = ± tan *θ*_1_/*q*, where *q* is the harmonic order, and *θ*_1_ is the half-cross angle of the two driving beams. The experimental divergence angles *θ*_1_ and *θ*_*q*_ were 64.9 mrad and 21.6 mrad, respectively, which satisfied the above divergence trend well with an error of less than 1 %. The nonlinear conversion efficiency of third harmonic generation was 1.6 × 10^−4^ %. We observed a third harmonic pattern at position (1) which was generated more clearly. Figure 5(b)–(d) show the generated ‘3’ pattern for the driving IR, SH-UV, and TH-DUV, respectively. The pattern size decreases as the harmonic order increases, which can be explained by the abovementioned three models: the wave model, the photon model, and the Fraunhofer diffraction theory. The spacing of dots was 48.7 μm for IR, 24.2 μm for SH-UV as aforementioned, and 15.1 μm for TH-DUV, which decreased almost linearly with wavelength. The graph on the right side shows the cross-sectional intensity distribution of the white dotted line. The scale of the *x*-axis was divided by the wavelength of a driving beam or harmonic waves. It was confirmed that the relative distribution of the pattern remained constant even when the harmonic order was increased. Meanwhile, in the case of the third harmonic, the pattern quality was degraded at the edge of the pattern, as shown in Figure 5(d). This is because the nonlinear polarization of the harmonic waves strongly depended on the intensity in higher orders. As the edge of the interference pattern at the crystal did not provide sufficient intensity to induce a third-order nonlinear polarization, the effective generation area for the third harmonics should have decreased from that for the second harmonic generation. Consequently, the pattern quality at the focal plane was slightly degraded.

## 4 Discussion

Our configurations for UV harmonic modulation are expected to provide useful functionality in diverse optical applications by allowing a change in the spatial distribution of UV light in real time and polarization resolution. Specifically, in terms of optical information processing and encryption, the amount of pixelated SH-UV information can be doubled or increased even more by tuning the polarization condition. Real-time beam control capabilities will enable active optical encryption with higher security, which is difficult to achieve through pre-designed elements. This real-time property also allows active optical trapping with a high degree of freedom, thus providing a potential tool for quantum engineering [28]. Additionally, our active UV beam shaping will be able to offer enhanced beam controllability by combining with the extensively reported metasurface or 2D structure-based beam control techniques [29–31]. The dual-foci concept has the potential to dynamically change the focus, indicating the feasibility of 3D holograms [32] in the UV regime. In particular, the MgO crystal used in this experiment can produce an ultrashort EUV pulse with higher-order harmonics, providing an effective EUV beam control for high-energy photon spectroscopy that requires attosecond-scale timing resolution [33].

Improving the resolution of harmonic UV patterns needs to be considered as an additional study. The resolution of a harmonic wave pattern is influenced by the pixels of the SLM. According to the spatial frequency term in Eq. (1), the resolution of the harmonic wave pattern, represented by the reciprocal of the spatial frequency, depends on the maximum achievable x value on the SLM. Currently, with *x* = 15,900 μm, the spatial resolution of the driving IR, SH-UV, and TH-DUV pattern is 10 μm, 5 μm, and 3.3 μm, respectively. The spatial resolution can be further enhanced by employing a lens with a shorter focal length. For instance, by utilizing a lens with *f* = 50 mm, the pattern resolution for the TH-DUV could reach 825 nm. These improved resolutions hold significant potential for more precise UV applications.

Further studies may include the calculation of the phase distribution of harmonic UV-DUV using the phase-retrieval algorithm. Generally, the phase of the generated harmonic wave can be shifted by intensity-driven phase mismatching of the driving beam [34]. Therefore, it is important to understand how the phase information of the driving beam is transferred to the harmonic waves on the surface of the nonlinear medium according to the harmonic order. In our experiment, as a higher intensity of the driving beam was used to generate a third harmonic, the degradation of the TH-DUV pattern could be attributed to the intensity-dependent phase mismatching term, in addition to the reduced effective generation area, as previously mentioned. To generate a clearer pattern for higher-order harmonics, these two effects must be considered. Phase-mismatching terms can be controlled using SLM [35]. The pattern quality of higher-order harmonics can be improved by including a compensated phase map in the SLM, which can reduce the phase mismatching effect. In addition, since third-order or higher harmonic waves are emitted in several directions by momentum conservation, controlling the separation of generated harmonics has been considered an important point. By employing counter-rotating circular polarization for the two driving beams, harmonic waves can be concentrated and emitted only at the minimum angle, *θ*_*q*_ [24]. With this polarization modulation, it will also be possible to selectively propagate our patterned harmonics. Alternatively, in order to increase the diffraction efficiency of the harmonic wave in a specific direction, an additional phasemap for forming a blazed grating on the focal plane can be input to the SLM. This phase-compensation and polarization modulation can realize the effective control of the spatiotemporal properties of high-order harmonic EUV pulses.

## 5 Conclusions

We have demonstrated the modulation of harmonic waves from UV to DUV regime using the programmable spatial phase modulation of the driving beam. The coherent frequency upconversion process allowed the transfer of spatiotemporal information of the driving beam to the harmonic UV pulses. The SLM-based driving beam modulation provides several advantages in UV beam shaping. First, it does not require specially designed or highly complex UV optics or nonlinear crystal. Second, the harmonic UV beam pattern can be controlled in a polarization-resolving manner in real-time. Third, the non-collinear harmonic generation permits the spatially shaped UV harmonic to be readily separated from the driving beam. This beam shaping of the third harmonic DUV offers meaningful insights into the control of higher-order EUV pulses. Our UV manipulation technique will enable arbitrary wavefront control of UV laser beams in applications like high-precision laser patterning, polarization-resolved encryption, and dynamic atom trapping.

## Supplementary Material

Supplementary Material Details
