# Supplementary material for: Dynamic ultraviolet harmonic beam pattern control by programmable spatial wavefront modulation of near-infrared fundamental beam
Source: Nanophotonics. 2023 Jun 23;12(16):3373–83. doi: 10.1515/nanoph-2023-0300 (PMC11501847; doi:10.1515/nanoph-2023-0300)
Supplement: Supplementary file 7 — Supplementary Material Details [file j_nanoph-2023-0300_suppl_001.docx]

Dynamic ultraviolet harmonic beam pattern control by programmable spatial wavefront modulation of near-infrared fundamental beam: Supplementary material

1. Interference pattern on the surface of a nonlinear medium

When a driving beam is incident on a nonlinear medium, the interference pattern produced on the surface can be represented by the following equation [1]:

$$I\left( x,y \right)=\left| E_{1}\left( x,y \right)+E_{2}\left( x,y \right) \right|^{2}=I_{0}\left( x,y \right)\left[ 1+\frac{A_{1}\left( x,y \right)A_{2}\left( x,y \right)\left( e_{1}\cdot e_{2} \right)}{I_{0}\left( x,y \right)}cos(k_{0}sin\theta x+\varphi_{1}\left( x,y \right)-\varphi_{2}\left( x,y \right)) \right],$$

$$E_{1}=A_{1}\left( x,y \right)exp \left( -j\varphi_{1}\left( x,y \right) \right) , E_{2}=A_{2}\left( x,y \right)exp \left( -j\varphi_{2}\left( x,y \right) \right) ,$$

$I_{0}=\frac{1}{2}\{A_{1}\left( x,y \right)^{2}+A_{2}\left( x,y \right)^{2}\}$ , S1

where $x$ and $y$ represent the Cartesian coordinates on the sample surface; $E_{1}(x,y)$ and $E_{2}\left( x,y \right)$are the complex amplitudes of driving Beams 1 and 2, respectively; $A_{1}\left( x,y \right)$and $A_{2}\left( x,y \right)$are the amplitudes of each beam; $\varphi_{1}\left( x,y \right)$and $\varphi_{2}\left( x,y \right)$are the phases of each beam; $I_{0}\left( x,y \right)$is the DC intensity term; $e_{1}$and $e_{2}$ are the linear polarization vectors; *θ* is the crossing angle between the two beams; $k_{0}(=\frac{2\pi}{\lambda})$ is the wavevector. This equation suggests that the interference pattern is affected by not only the crossing angle but also the complex amplitude of each beam.

1. Phase matching condition in non-collinear harmonic generation

The phase matching condition in non-collinear harmonic generation can be expressed by the following equation [2]

$\Delta k=q_{1}k_{1}\cos\left( \theta_{1}-\theta_{q} \right)+q_{2}k_{2}\cos\left( \theta_{2}-\theta_{q} \right)-k_{q}+\Delta k_{geo}+\Delta k_{atom}+\Delta k_{elec}-\theta\nabla I$*,*

S2

where $\Delta k$ is the phase mismatch that should be minimized for efficient harmonic generation. $k_{1}$,$k_{2}$, and $k_{q}$ are the wave vectors of the two driving beams and the q-th harmonics. $\theta_{1}$,$\theta_{2}$, and $\theta_{q}$ are the propagating angles of two driving beams and the q-th harmonics. $\Delta k_{geo}, \Delta k_{atom}, \Delta k_{elec},$and $\theta\nabla I$ are geometric dispersion known as Gouy phase, atomic dispersion, electronic dispersion, and atomic dipole phase, respectively. These terms are factors that cause phase mismatching and reduce harmonic efficiency. In the non-collinear harmonic generation with a tightly focused driving beam, the atomic dipole phase is a main factor inducing phase mismatching because it depends on the gradients of driving laser intensity [2]. In the case of a sparse gas medium, the propagation vector of harmonics generated before the focus is distorted since the vector of the dipole phase has a radial direction. On the other hand, in the solid medium, phase mismatching including atomic dipole is negligible because the interaction layer is relatively shorter than the gas due to strong reabsorption [3].

1. Momentum conservation of photons in non-collinear harmonic generation

Ignoring the phase mismatching term, the momentum conservation of two photons in non-collinear harmonic generation can be expressed by the following equation

$k_{q\left( x,y \right)}=q_{1}\left( x,y \right)k_{1}\left( x,y \right)+q_{2}\left( x,y \right)k_{2}\left( x,y \right),$ S3

where $k_{1}$ and $k_{2}$represent the wavevectors of driving Beams 1 and 2, respectively; $k_{q}$ is the wavevector of the *q*-th harmonic beam; $q_{1}$ and $q_{2}$ indicate the number of photons contributed by driving beams 1 and 2 to the harmonic generation, respectively. The harmonic order q is equal to the sum of the number of input photons (*q* = $q_{1}$ + $q_{2}$). In the case of second harmonics, there are three possible sets for ($q_{1}$_,_$q_{2}$), that is, {(0,2),(2,0),(1,1)}. In the case of (1,1), in which each photon $q_{1}$_,_ $q_{2}$ is identically involved, the generated second harmonic propagates with half of the angle between the two driving beams.

1. Output power and conversion efficiency of UV harmonics

The conversion efficiency of harmonic waves can be defined as follows [4].

$\eta(Conversion efficiency)=\frac{\mathrm{Output} \mathrm{power} \mathrm{of} the h\mathrm{armonics} (W)}{\mathrm{Input} \mathrm{power} \mathrm{of} the \mathrm{driving} \mathrm{beam} (W)}\times100 (\%)$ S4

We measured the output power and conversion efficiency of the 2^nd^ and 3^rd^ harmonics. The total power of the two driving beams immediately before the focusing lens was measured using a thermal power sensor (30(150)A-BB-18, Ophir), and the power of harmonic waves was measured using a silicon photodetector (PD10-C, Ophir). The driving IR beam was blocked by stainless steel plate. Table S1 shows the power and conversion efficiency of UV harmonics under various conditions. Since nonlinear susceptibility decreases as the harmonic order increases [5], the conversion efficiency of the third harmonics was one order lower than that of the second harmonics. In addition, in the case of modulated harmonics (generating a harmonic wave via driving beam modulation using SLM), the efficiency was about 9% to 20% lower than that of an unmodulated harmonic wave. This is because the power distribution of modulated beam is widely scattered due to phase manipulation and does not completely overlap with the 0th-order beam in the nonlinear medium. On the other hand, when the polarizations of the two driving beams are perpendicular to each other, the output power of harmonics was reduced (9% for 2^nd^ harmonics and 31% for 3^rd^ harmonics) compared to the case of the same polarized driving beams. Under the same polarization condition, the peak intensity increases due to interference, so the conversion efficiency can be increased. However, when driving beams of orthogonal polarizations are used, the power of harmonics cannot be increased because interference does not occur. In particular, in the case of the 3^rd^ harmonic wave, which is more dependent on intensity of the driving beam, there is a large power difference depending on the polarization of the two driving beams. One potential strategy to enhance conversion efficiency may include setting the overlapping position of two driving beams closer to the focal point, thereby increasing the intensity of the driving beam incident on the nonlinear sample.

|  | | Driving  Beam power  (mW) | UV harmonic power (μW) | Conversion efficiency  (%) |
| --- | --- | --- | --- | --- |
| 2^nd^ harmonic  from quartz  (λ=400 nm) | 0^th^-order beam | 155.65 | 2.05 | 1.3×${10}^{-3}$ |
|  | Modulated beam |  | 1.57 | 1.0×${10}^{-3}$ |
|  | 0^th^-order beam with orthogonal polarization |  | 1.86 | 1.2×${10}^{-3}$ |
| 3^rd^ harmonic  from MgO  (λ=266 nm) | 0^th^-order beam | 273.58 | 0.48 | 1.7×${10}^{-4}$ |
|  | Modulated beam |  | 0.44 | 1.6×${10}^{-4}$ |
|  | 0^th^-order beam with orthogonal polarization |  | 0.33 | 1.1×${10}^{-4}$ |

Table S1. Output power and conversion efficiency of 2^nd^ and 3^rd^ harmonics.

1. Effect of the power ratio of the two driving beams on the UV pattern image

Depending on the power ratio of the two driving beams, the properties of the generated UV pattern are affected. Figure S1 shows SH-UV images according to the power ratio (Beam 1/Beam 2) of the two driving beams. Beam 1 is a phase-modulated driving beam, and beam 2 is an un-modulated driving beam. The total power sum of Beam 1 and Beam 2 was set equal for each condition. In order from the left, images are shown at power ratios of 0.02, 0.42, 1.03, 3.84, and 15.45. When the power ratio of the two driving beams was almost 1, the brightest UV pattern was created. However, when there was a large power difference between the two driving beams, the intensity and quality of the UV pattern decreased.


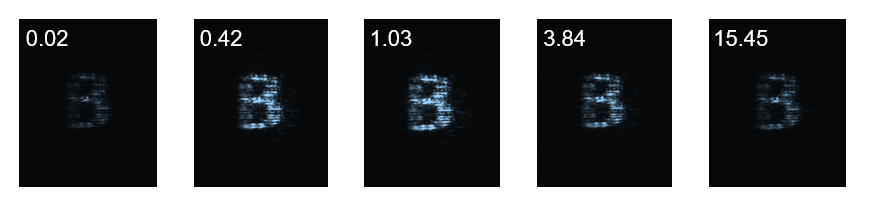


Figure S1. SH-UV image according to the power ratio of two driving beams (Beam 1/Beam 2).

1. Effect of the geometrical alignment on output power and pattern quality of UV harmonics

The geometrical alignment can affect the harmonic yield and quality of the modulated pattern. Figure S2 shows the normalized output power and pattern images of SH-UV according to the geometrical alignment of the quartz sample. The output power or pattern quality of the harmonic wave was not significantly impacted by the tilt angle of the sample because the solid medium is less affected by the phase matching condition (Figure S2(a)). On the other hand, the power of UV harmonics or the quality of the pattern reduced depending on where the sample was placed on the optical axis (Figure S2(b)). In order to ensure that all driving photons participate in the harmonic generation, the nonlinear sample must be placed at the exact position (0 mm in Figure S2(b)) where the two driving beams entirely overlap. However, even slight axial displacements of the sample by a few millimeters along the z-axis substantially diminish the overlapped region of the two driving beams, leading to a notable decrease in harmonic power and a degradation in pattern quality.


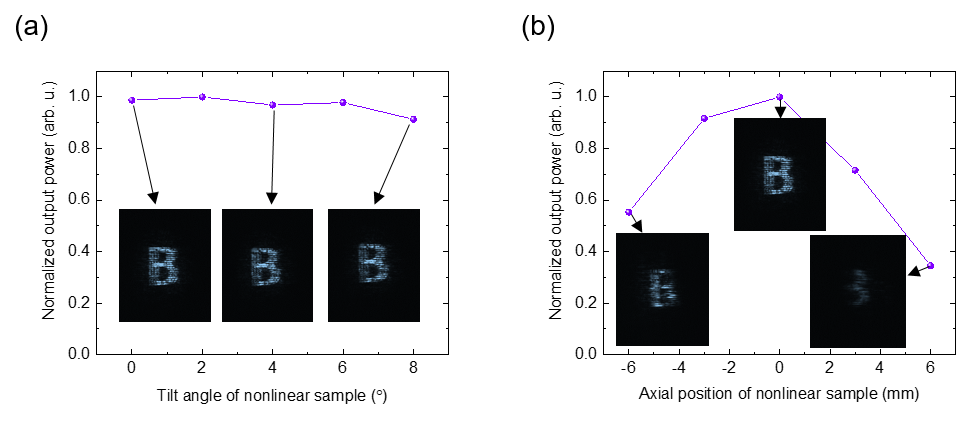


Figure S2. Normalized output power and pattern images of SH-UV according to the geometrical alignment of the nonlinear sample. (a) Tilt angle of the sample. (b) Axial position of the sample.

REFERENCES

[1] G. M. Burrow, T. K. Gaylord, "Multi-beam interference advances and applications: nano-electronics, photonic

crystals, metamaterials, subwavelength structures, optical trapping, and biomedical structures," Micromachines, vol.

2, pp. 221-257, 2011.

[2] T. Brabec, "Strong Field Laser Physics," Springer, New York, 2009.

[3] B. Kim, S. Choi, Y. W. Kim, S. J. Won, Y.-J. Kim, S.-W. Kim, "Optics-less beam control of EUV high harmonics generated from solids," arXiv:2111.14085, 2021.

[4] S. Kurimura, M. Harada, K. Muramatsu, M. Ueda, M. Adachi, T. Yamada, T. Ueno, “Quartz revisits nonlinear

optics: Twinned crystal for quasi-phase matching [Invited],” Opt. Mat. Exp. Vol 1, p. 1367, 2011.

[5] S. Han, L. Ortmann, H. Kim, Y. W. Kim, T. Oka, A. Chacon, B. Doran, M. Ciappina, M. Lewenstein, S.-W. Kim,

S. Kim, A. S. Landsman, "Extraction of higher-order nonlinear electronic response in solids using high harmonic

generation," Nat. Commun. vol. 10. p. 3272, 2019.
